# Gamification and the development of metacognitive processes: a systematic literature review

**DOI:** 10.12688/f1000research.173134.1

**Published:** 2025-12-10

**Authors:** Claudia Viviana Varela Quiroga, Mauricio Esteban Buitrago Ropero

**Affiliations:** 1 Cundinamarca, Universidad Antonio Narino, Bogotá, Cundinamarca, Colombia; 2 Cundinamarca, Fundacion Universitaria Los Libertadores, Bogotá, Cundinamarca, Colombia

**Keywords:** gamification, metacognition, cognitive skills, systematic review

## Abstract

**Background:**

Gamification has encouraged growing interest in educational research because of its potential to improve motivation, participation, and self-regulated learning. However, its contribution to the development of metacognitive processes has not been studied thoroughly. This systematic literature review examines how gamification-based strategies support metacognitive processes, such as planning, monitoring, and evaluation, in educational contexts (García-López et al., 2023; Gkintoni et al., 2024).

**Methods:**

This review was conducted using the PRISMA 2020 methodology. Searches were conducted in Scopus and the Web of Science using the terms gamification, education, and metacognition. Only peer-reviewed, open-access articles published between 2019 and 2024 were included. Inclusion and exclusion criteria were applied during the selection of titles and abstracts and during the full-text review. After removing duplicates (n = 115), 253 records were examined; 181 were excluded because of irrelevance and 71 were included in the final analysis. Thematic analysis was used to extract information on theoretical frameworks, methodologies, metacognitive components, and higher-order cognitive processes.

**Results:**

The findings revealed that gamification positively contributes to metacognition by activating processes, such as self-regulation, monitoring, and decision-making. The central elements of gamification, such as immediate feedback, progressive challenges, autonomy, and reward systems, support metacognitive awareness, and encourage active participation. Frequently cited theoretical frameworks, including studies, show that gamification promotes higher-order thinking skills such as problem-solving and critical thinking.

**Conclusions:**

Gamification strengthens metacognitive processes when instructional design integrates meaningful goals, feedback mechanisms, cognitive challenges, and reflection opportunities. Evidence suggests that educational environments based on gamification are effective in promoting the planning, monitoring, and evaluation skills essential for autonomous learning and metacognitive development ( Ask et al., 2023). Future research should expand the scope of these databases and include non-open-access studies to reduce potential biases.

## 1. Introduction

In recent decades, educational models have evolved toward approaches focused on students, promoting active methodologies aimed at meaningful learning ( Edo Agustín, 2023). Gamification has emerged as an innovative pedagogical strategy with the potential to positively impact the development of metacognitive processes by fostering students’ awareness of their own learning processes and promoting autonomy, self-reflection, and self-regulation.

Over the last five years, academic production has shown significant interest in understanding how gamified dynamics influence the development of higher-order thinking skills, such as metacognition ( Beça et al., 2022; Dong et al., 2024; Pirta-Dreimane et al. 2024). Studies by Rosli et al. (2019) and Cáceres Reche et al. (2022) highlighted that gamification favors individual learning, fosters cooperation and social awareness, and facilitates collaborative learning environments.

This study presents a Systematic Literature Review (SLR) aimed at analyzing the relationship between gamification and metacognitive processes in educational contexts. This study focuses on gamification based on cognitive theories that encourage critical and conscious reflection. Gamification is understood as the application of the characteristic elements of games, such as rewards, challenges, immediate feedback, and autonomy, in non-playful contexts to promote intrinsic motivation for learning ( Hwang et al., 2024; Leitão et al., 2022). Metacognition is the ability to reflect on one’s own cognitive process. This implies an advanced level of awareness and voluntary control over learning, which allows for the planning, monitoring, and evaluation of mental activities ( Antonaci et al., 2019; García-López et al., 2023). Thus, the development of these metacognitive processes is essential for problem-solving and the effective transfer of knowledge to new contexts.

Likewise, the relationship between gamification and metacognition has been highlighted by authors, such as Peuters et al. (2024), who argued that rewards and the mechanisms of self-regulation embedded in gamification can activate metacognitive processes such as planning and monitoring. Self-regulation is a learner’s ability to plan, monitor, and adjust learning strategies autonomously in response to challenges and feedback and is essential for achieving meaningful learning ( Zhao et al., 2024). Similarly, Yáñez De Aldecoa and Gómez-Trigueros (2022) argued that gamification enables students to analyze, reflect on, and adjust their learning strategies.

According to Yáñez De Aldecoa and Gómez-Trigueros (2022), gamification allows students to analyze, reflect, and adjust their learning strategies. Thus, the relationship between gamification and metacognition can be understood through constructivist and self-regulated learning theories. Gamified environments promote active engagement and self-monitoring, which are the essential components of metacognitive processes. For instance, when learners face gamified challenges that provide immediate feedback, they are encouraged to reflect on their strategies, thereby reinforcing their metacognitive processes (planning, monitoring, and evaluation).

Despite this evidence, the implementation of gamification in educational institutions has hardly been explored, particularly regarding the analysis and study of its psychological and pedagogical frames, as well as its specific effect on the development of metacognition. This systematic review examined the use of gamification to strengthen metacognition with an emphasis on its pedagogical implementation.

## 2. Materials and Methods

This study was developed following the PRISMA model guidelines (Preferred Reporting Items for Systematic and Meta-Analyses) ( Moher et al., 2009) publications indexed between January 2019 to November 2024 focusing on the relationship between gamification and metacognitive processes in educational contexts were selected; the objective was to answer the following research questions:

RQ1. What is the relationship between gamification use and metacognitive processes?

RQ2. What theoretical frameworks support the use of gamification in fostering the development of metacognitive processes in educational contexts?

RQ3. How is gamification related to the development of higher-order thinking skills in the higher education and educational contexts?

The search strategy was implemented using two databases: Scopus and Web of Science (WoS). A systematic search was conducted in these databases using the following search equation on November 2 ^nd^, 2024, covering publications from 2019 to 2024: only peer-reviewed, open-access, full-text academic articles written in English or Spanish were considered. The full-search equations are presented in Table 1.

**Table 1.** Search equation result.

Search equation	WoS	Scopus	Total
(Gamification) AND (Education) AND (“Cognitive Ability” OR Metacognition or Awareness)	203	165	368

***Note:*** Own elaboration.

This table presents the search equation applied in Web of Science and Scopus, along with the number of records retrieved from each database during the initial identification phase of the systematic review.

The search yielded 368 documents (203 WoS and 165 Scopus). Subsequently, inclusion and exclusion criteria were established, as detailed in Table 2, considering aspects such as the year of publication, type of document, access to full text, thematic relevance in relation to the research questions, lack of metacognitive focus, lack of gamification in an educational context, and absence of full text access.

**Table 2.** Inclusion and exclusion criteria used in the systematic review.

Criteria	Inclusion	Exclusion
Year of publication	2019-2024	Before 2019
Type of document	Scientific articles	Doctoral theses, book chapters
Access	Open access	Inaccessible documents
Relevance	Answer at least two questions	Answer only one question

***Note:*** Own elaboration.

This table summarizes the criteria used to determine the eligibility of studies, detailing the conditions required for inclusion and the reasons studies were excluded during the screening and full-text assessment phases.

The study was performed by two independent reviewers who screened titles, abstracts, and keywords based on the inclusion and exclusion criteria. All records were screened by two reviewers during all the phases of the selection process. Discrepancies were discussed between the reviewers regarding the inclusion or exclusion of studies, and resolved by consensus. A third reviewer was not required.

The selection process was structured in three phases, according to the PRISMA model:

**Identification:** A total of 368 documents were retrieved from the two databases using the search equation, of which 115 duplicate records were removed, leaving 253 documents for the next phase.

**Screening:** A preliminary review of titles, abstracts, and keywords was conducted by applying thematic relevance criteria; 181 articles were excluded in this phase to avoid directly addressing research questions.

**Deep reading:** The remaining seventy-two articles were analyzed in a matrix. In this stage, aspects such as the theoretical framework, applied methodology, results, main findings, and relevance of the study’s questions were analyzed. During this phase, one article was excluded after verifying that it had been reported as plagiarized, because it was not part of the corresponding database. The exclusion criteria were ethical and scientific considerations were excluded from this study. Seventy-one final studies were included in the thematic analysis.

Figure 1 presents a PRISMA flow diagram summarizing the study-selection process.

**Figure 1.**
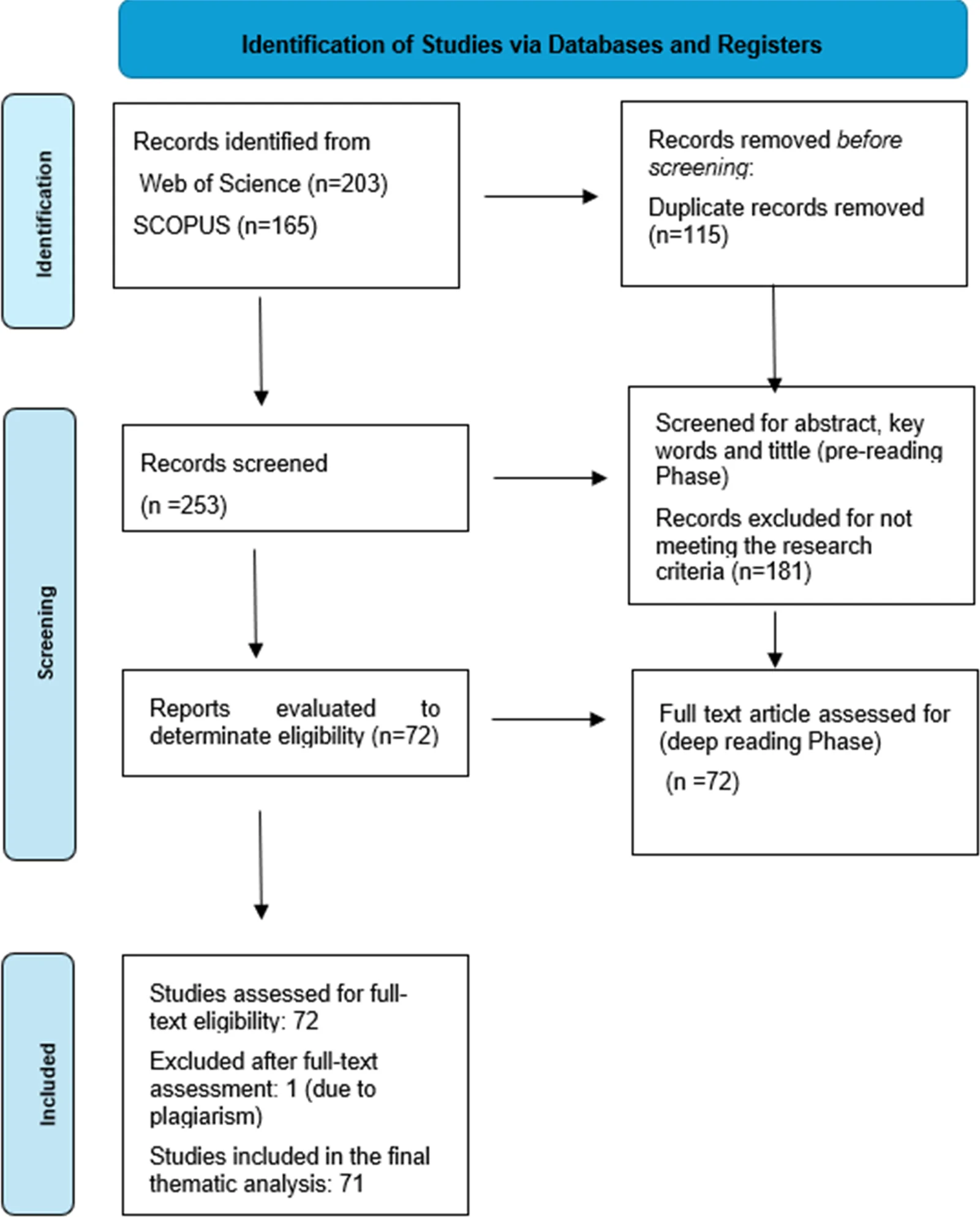
Flow diagram of the selection process according to the PRISMA model. This figure illustrates the identification, screening, eligibility assessment, and inclusion of studies following the PRISMA 2020 guidelines, showing the number of records identified, excluded, and retained for the final analysis.

Data extraction was performed by two independent reviewers using a predefined data-extraction matrix developed in Excel. The following samples were extracted from each study: general bibliographic information, study-level metadata, methodological characteristics, gamification-related variables, metacognition-related variables, theoretical frameworks, higher-order cognitive skills, relevance to each research question, and main conclusions extracted from each study. These items ensured a detailed and comparable extraction across all studies, supporting transparency and the synthesis process.

The risk of bias in the studies was evaluated using criteria adapted from qualitative evidence. Two reviewers evaluated each study for clarity of objectives, suitability of design, transparency, rigor of analysis, and ethical considerations. Disagreements were resolved through consensus. Similarly, no data conversion was required because the qualitative nature of the synthesis and publication bias could not be statistically evaluated, owing to the absence of quantitative synthesis.

## 3. Results

The results were presented based on the answers and findings obtained in relation to the research questions.

### RQ1. What is the relationship between gamification and the development of metacognitive processes?

The findings show a meaningful relationship between the gamification process and the development of metacognitive processes such as monitoring and self-regulation of learning. Antonaci et al. (2019), Goddiksen et al. (2024), and Mei and Yang (2019) agree that elements of gamification such as immediate feedback, rewards, challenges, and autonomy foster critical reflection on learning and enhance students’ awareness of their cognitive performance.

According to Edo Agustín (2023), gamification generates favorable conditions for the construction of meaningful learning by promoting increased engagement with instructional content. This interaction is not merely superficial; by integrating strategies such as feedback, students can analyze their decisions and consciously correct errors, thereby enhancing critical reflection and logical thinking ( Hwang et al., 2024). However, this effect enables learners to move beyond unstructured trials and error dynamics are often observed in poorly designed gamified experiences.

An example of this is provided by Zhao et al. (2024), who emphasized that the use of augmented reality in gamified environments fosters reflection on one’s own learning, which in turn increases confidence in the use of technology. From this perspective, it can be stated that gamification activates internal self-regulation mechanisms, as it enables students to assess their progress, identify errors, set goals, and adjust cognitive strategies. This contributes to the development of personalized educational experiences and encourages self-assessment as an active component of the learning process.

Similarly, Beça *et al.* (2022) emphasized that the appropriate integration of game-based content enables students to achieve meaningful learning outcomes, such as winning, losing, and receiving feedback, which increases their awareness of the learning process. From this perspective, gamification extends beyond motivation and acts as a mediator in reflective thinking. Other studies, such as that of Cáceres Reche et al. (2022), have demonstrated how games with reward systems promote processes such as planning and monitoring. Similarly, Reche *et al.* (2022) highlight the academic, emotional, and social benefits of gamification. Emotionally, it enhances students’ motivation and interest in task completion while socially emphasizing its contribution to collaborative work by fostering environments that stimulate self-regulated learning.

Several studies, including Manshoven and Gillabel (2021) and Qiao (2024), indicate that gamified processes are widely related to emotional abilities such as autonomy, self-efficacy, and competence, which can be developed through well-designed gamification. Dong et al. (2024) highlighted that educational games that stimulate planning and problem solving are useful for consolidating significant and reflective learning.

In general, review studies such as those by Goddiksen et al. (2024) and Rosli et al. (2019) agreed that gamification enhances cognitive structures and fosters the development of factual knowledge, including memory and the acquisition of critical application skills in real-world scenarios. This reinforces the role of gamification as a pedagogical process that facilitates activation and enhancement of metacognitive processes.

### RQ2. What theoretical frameworks support the use of gamification to foster the development of metacognitive processes in educational contexts?

The literature review identified three fundamental theoretical categories that supported the frameworks used to explain the relationship between gamification and metacognitive processes. These categories are linked to learning methods, psychological theories, and underlying cognitive processes involved in teaching and learning. Together, these dimensions offer a comprehensive approach to understanding how gamification influences the development of metacognition in educational contexts by integrating a theoretical-practical perspective.

Table 3 summarizes the findings associated with each category, as reported in the analyzed studies. This quantitative perspective helps to identify the most commonly used foundations to justify the incorporation of gamification as a facilitator for developing the metacognitive process.

**Table 3.** Theoretical frameworks supporting the use of gamification in educational contexts.

Theoretical frameworks	Associated categories	Articles	Percentage
**Learning Methodologies**	Game-Based Learning (GBL)	43	60.6%
	Not specified	28	39.4%
**Psychological Theories of Learning**	Self-Determination Theory	3	4.1%
	Self-Regulated Learning Theory	6	8.5%
	Piaget’s Theory of Cognitive Development	1	1.4%
	Not specified	61	86%
**Cognitive Processes**	Self-Regulated Learning	7	9.9%
	Meta-Reasoning Model	5	7%
	Metacognition (explicitly mentioned)	1	1.4%
	Not specified	58	81.7%

***Note.*** Own elaboration.

This table synthesizes the theoretical categories identified across the reviewed studies learning methodologies, psychological theories, and cognitive processes and reports the number and percentage of articles associated with each category.

Regarding the methodologies used to implement gamification processes that foster metacognitive development, the literature points to Game-Based Learning (GBL) as the predominant approach. GBL was present in 60.6% of the articles analyzed, while the remaining percentage did not mention a specific methodology. Game-Based Learning is based on the incorporation of game dynamics within educational contexts with clearly defined learning objectives. According to Leal Uhlig et al. (2023), technology plays a key role in GBL environments as it facilitates immersive experiences that promote student engagement and generate positive impacts on the development of critical thinking. Likewise, Davis (2022) and Hwang *et al.* (2024) emphasized that the game-based approach increases intrinsic motivation and strengthens metacognitive processes, such as planning, monitoring, and self-reflection, by integrating disciplinary content with dynamic structures that stimulate conscious decision-making and the continuous evaluation of learning.

Davis (2022) explained that serious game-based learning is supported by Piaget’s cognitive theory, which integrates playful elements into instructional content, thereby improving motivation and learning. Game-based learning contributes to the development of cognitive processes such as reading comprehension, attention, and memory by combining cognitive theory, instructional content, and game elements that leverage the motivational and immersive nature of games to improve learning outcomes. In terms of metacognition, serious games promote planning, which is understood as the learner´s ability to anticipate actions and set goals before starting a task, and monitoring, which involves constantly reviewing and evaluating one´s own performance and understanding during the activity. These dynamics require reflection on the decisions made to allow for the adjustment of strategies based on the feedback received, thereby strengthening self-regulation of learning.

Game-based learning, supported by learning theories such as constructivism, favors the development of metacognitive processes by creating interactive environments that foster self-reflection and learning regulation. According to Hwang *et al.* (2024), GBL facilitates problem solving through active experimentation, allowing students to analyze decisions and receive immediate feedback. This process of analysis and decision making strengthens self-regulation and metacognitive awareness, as it requires constant evaluation of their strategies and adjustment according to the results obtained.

Dong *et al.* (2024) highlighted its application to students with cognitive disabilities, emphasizing that gamification improves skills such as memory, attention, reasoning, and planning. These findings show that game-based learning promotes the development of metacognitive strategies, such as organization, adaptation to new situations, and reflective decision-making, all of which are essential for autonomy in inclusive educational environments.

The literature review makes it possible to identify that, from a psychological perspective, three fundamental theories support the learning process: the theory of self-regulated learning, self-determination theory, and Piaget’s theory of cognitive development. The first was the theory of self-regulated learning, which was presented in 8.5% of the reviewed articles. This theory is presented as a psychological foundation, as it analyzes how students plan, monitor, and evaluate their learning process. In gamified contexts, these processes are enhanced by dynamics such as immediate feedback, personalized challenges, and progress monitoring (Kim et al. 2021, as cited in Choi et al., 2022). According to Marks et al. (2024), gamification fosters a self-regulated mind-set by facilitating the identification of knowledge gaps and strategic adjustments through constructive feedback.

Figure 2 illustrates the distribution of the articles according to the identified theoretical frameworks.

**Figure 2.**
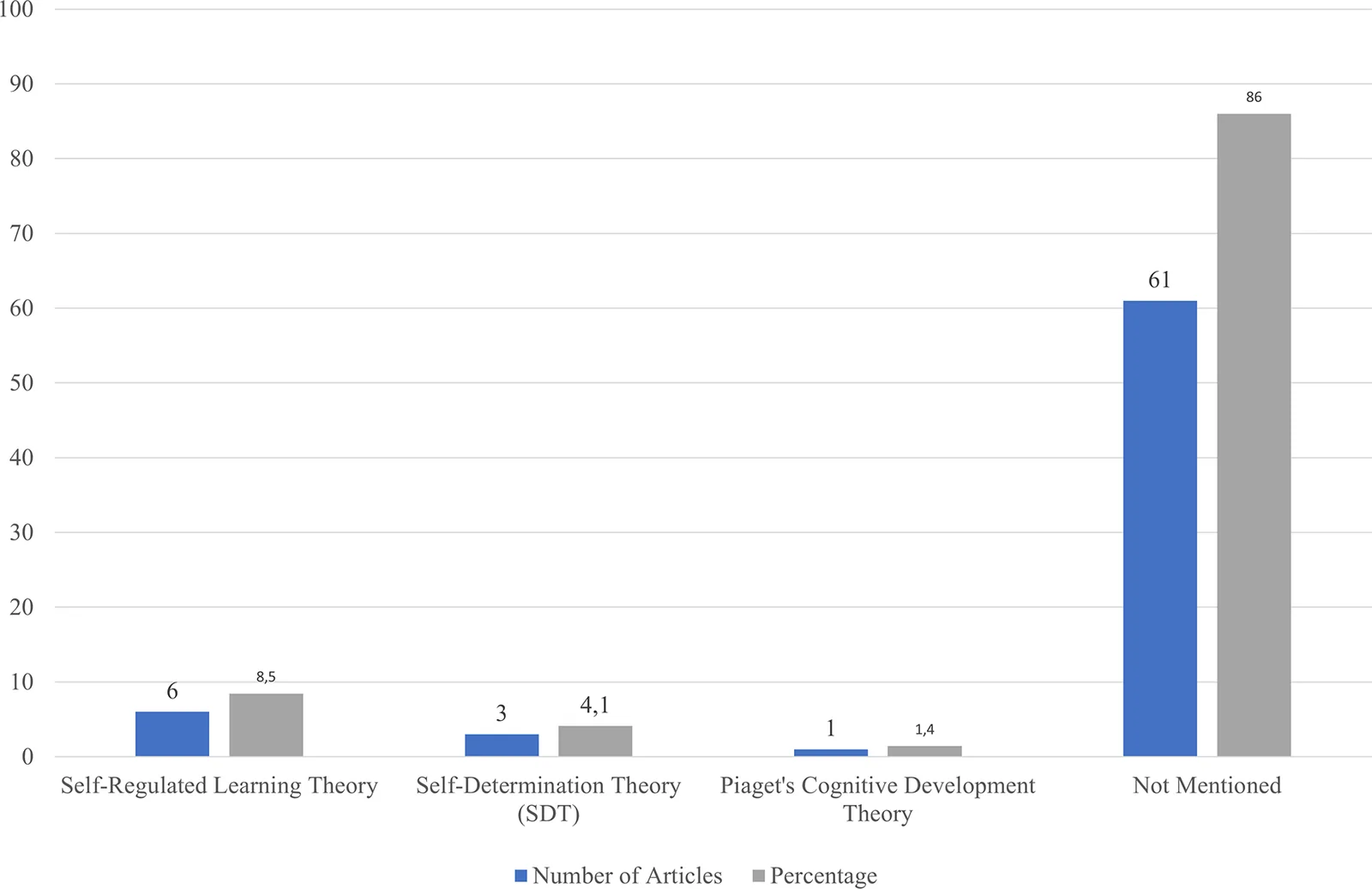
Articles based on theories. This figure presents the number and percentage of studies in the systematic review classified by their underlying theoretical foundation, highlighting the predominance of studies that did not explicitly mention a guiding theory.

The second theory identified is Self-Determination Theory (SDT), which was presented in 4.2% of the articles analyzed. This theory, proposed by ( Deci & Ryan, 2000), emphasizes the importance of intrinsic motivation, which arises from interest in and enjoyment of the activity itself and is essential for promoting meaningful learning. SDT states that this form of motivation is activated when three basic psychological needs are satisfied: autonomy, understood as the student’s ability to make decisions and act with a sense of regulation; competence, referring to the feeling of effectiveness when facing challenges; and social relatedness, which implies a feeling of connection with others and establishment of meaningful relationships ( Qiao et al., 2022).

In gamified environments, these needs are activated through specific mechanics, such as avatar customization (autonomy), progressive difficulty levels and achievements (competence), and collaborative interactions (relatedness). Satisfying these needs enhances students’ engagement and willingness to reflect on their learning, thereby promoting metacognitive processes ( Calderón et al. 2024; Wan et al. 2021; Siala *et al.* 2019).

The third theory is Piaget’s Theory of Cognitive Development, which was cited in 1.4% of the reviewed articles. This provides a fundamental framework for understanding how gamification can favor both cognitive development and metacognitive processes in educational settings. Piaget postulated that children learn through evolutionary stages related to functional, constructive, symbolic, and rule-based play, which plays a decisive role in the development of emerging cognitive abilities ( Dong et al. 2024). Studies, such as those by Dong *et al.* (2024), have designed gamified experiences for children with cognitive impairment, showing that adapting playful mechanics to their level of development through positive feedback activities and progressive challenges improves functions such as memory and reasoning.

Likewise, *Piaget’s Theory of Cognitive Development* posits that knowledge is built through active interaction between the subject and environment. Gamification, by structuring playful dynamics with learning objectives, allows students to progressively construct and reconstruct their knowledge. These findings extend to the development of metacognitive skills, since gamification, by integrating reflective strategies, such as self-assessment journals and evaluation spaces, promotes awareness of one’s own learning processes. For example, students in gamified environments report their ability to monitor progress, adjust strategies, and reflect on their understanding ( Trigueros et al., 2024).

In contrast, within the psychological theories underlying the analysis of gamified processes in relation to metacognition, three main approaches were identified: self-regulated learning, metacognition, and the meta-reasoning model. Figure 3 shows the articles based on a single cognitive process.

**Figure 3.**
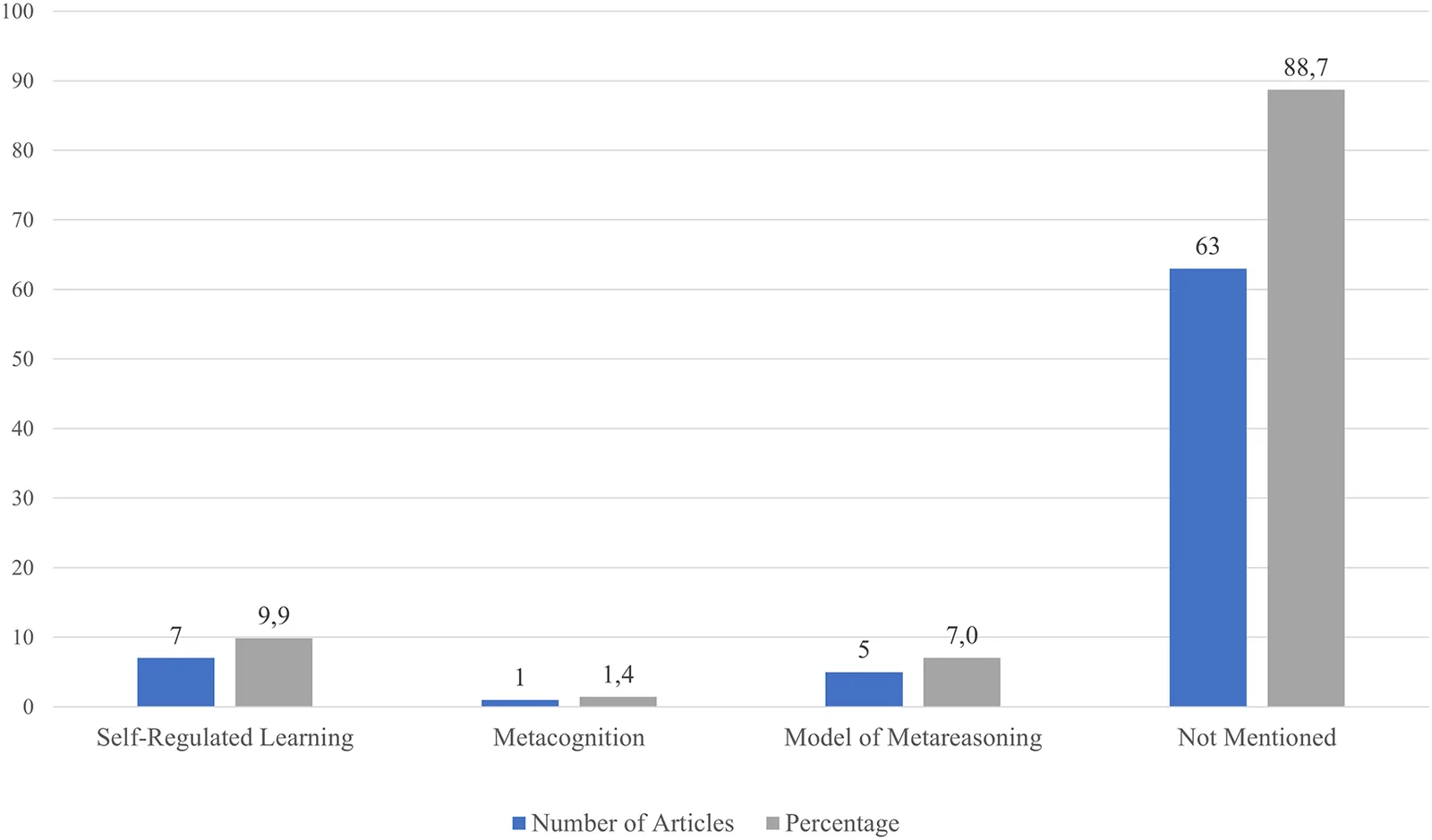
Articles based on cognitive processes. This figure shows the number and percentage of studies categorized according to the cognitive or metacognitive processes addressed, emphasizing the limited explicit focus on metacognitive frameworks.

Self-regulated learning is recognized as an effective strategy for mastering knowledge and improving attitudes toward learning ( Zhao et al., 2024). This perspective emphasizes students’ management of their thoughts, emotions, and behaviors to achieve their goals, thus promoting an active role through planning, monitoring, and adjusting personal strategies. Self-regulated learning appears in 9.9% of the studies, reaffirming its role as a bridge between gamification and the development of metacognitive processes.

Similarly, self-regulated learning enables learners to develop self-determination and reach a state of active awareness in their activities, characterized by important levels of concentration and motivation. Studies such as those by Manshoven and Gillabel (2021); Trigueros et al. (2024) confirm that gamification, by designing environments that meet these fundamental needs, fosters self-determination and active engagement with learning tasks. This framework explains how elements such as immediate feedback systems, level progression, and collaborative dynamics motivate and create ideal conditions for the development of metacognitive processes, by turning students into conscious agents of their learning processes.

Another cognitive process identified was the meta-reasoning model, which was cited in 7% of the articles. This model focuses on monitoring mental processes during decision making, in contrast to metacognition, which supervises the entire learning process. According to ( Kleitman & Narciss, 2019), meta-reasoning examines how individuals assess the difficulty of a task, and how these evaluations determine the amount of time, effort, and cognitive resources they are willing to invest in. Gamified contexts are applied through games that require problem-solving, error analysis, and strategic reflection. These dynamics enable students to make decisions and evaluate tasks, thereby contributing to the development of self-regulation and effort control ( Juan-Lázaro & Area-Moreira, 2021). On the other hand, metacognition as an explicit construct was mentioned in only 1.4% of the articles, suggesting that, although it is often the ultimate goal of gamified interventions, it is frequently addressed in an indirect or implicit manner.

In summary, the analyzed theoretical frameworks support the use of gamification as a mediator for developing metacognition in educational contexts. This perspective emphasizes the internal processes of learning, such as motivation, self-regulation, and reasoning, in line with Juan-Lázaro and Area-Moreira (2021). In a pedagogical framework, the focus is on designing meaningful student-centered experiences. Both perspectives agree that beyond motivating, gamification promotes conscious reflection on learning, which is essential for fostering autonomous and meaningful learning ( Voreopoulou et al., 2024).

### RQ3. How is gamification related to the development of higher-order thinking skills in educational contexts?

The reviewed studies establish a significant relationship between gamification and the development of Higher-Order Thinking Skills (HOTS) in educational settings ( Laksana et al., 2024). These skills encompass processes such as analyzing, evaluating, creating, problem-solving, and decision-making. This review highlights problem solving, critical thinking, and decision-making as the most prominent ( Qiao, 2024).

According to Edo Agustín (2023), the relationship between the use of gamification and the development of metacognitive processes in learning is enhanced when gamification incorporates explicit mechanisms that promote conscious reflection on the learning process as it offers immediate feedback and invites students to face real situations that demand complex cognitive processes. Likewise, gamification promotes the development of higher-order skills by going beyond the repetition of knowledge; it favors analysis, synthesis, critical, reflective, logical thinking, problem-solving, and the ability to create ( Hsbollah & Rosli, 2022). Antonaci *et al.* (2019) indicated that gamified environments promote higher-order thinking skills by requiring demanding cognitive abilities such as problem-solving, decision-making, and critical analysis, in challenging contexts that stimulate deep reflection.

However, the development of higher-order thinking skills is consciously and effectively presented when gamification is implemented through digital technologies, accompanied by intentional instructional design. Thus, elements such as immediate feedback, cognitive challenges, and the possibility of trial and error can activate advanced cognitive processes ( Hwang et al., 2024). According to Voreopoulou *et al.* (2024), the role of emerging technologies such as augmented reality shows that gamified activities involve exploration, analysis, and evaluation, which makes higher-order thinking skills (HOTS) more explicit in the learning process. Similarly, Calderón et al. (2024) affirmed that solving challenges requires the application of thinking and logical skills (cognitive challenges), and often overcoming challenges that depend on solving previous situations, which implies a progression in the complexity of thinking.

Based on these findings, Oliva et al. (2021) pointed out that strategic decision making is a constant demand in gamified environments, in which students must define paths to achieve objectives. When gamification and metacognition are articulated, students make decisions, think about strategies, evaluate different options, and learn from their responses. Similarly, they self-assess their processes, which promotes critical thinking and planning skills.

In general, the relationship between gamification and metacognition is directly associated with the development of higher-order skills ( Pirta-Dreimane et al., 2024), since both approaches promote problem-solving, critical thinking, self-regulation, planning, and monitoring, especially through challenging scenarios that stimulate high-level thinking.

## 4. Discussion

This systematic review explored the existing literature in relation to the influence of gamification on the development of metacognitive processes in educational settings; the analysis of the included studies shows a significant relationship between the use of gamification and the strengthening of metacognitive skills, especially those associated with self-regulation, planning, monitoring, and evaluation of one’s own learning ( García-López et al., 2023; Peuters et al., 2024).

According to the reviewed studies, gamification increases students’ commitment to learning by creating their own routes to reaching goals through metacognitive processes, and it is emphasized that gamification promotes the application of knowledge and the ability to face complex cognitive processes ( Pirta-Dreimane et al., 2024). Elements, such as feedback, progressive challenges, immediate rewards, and constant reflection stand out. These stimulate metacognitive processes and allow the establishment of learning objectives and strategies in a structured and conscious manner ( Blain et al. 2022; Zhao et al. 2024).

Similarly, the incorporation of gamified processes into educational settings has proven to be an effective pathway for fostering motivation, engagement, critical thinking, and the awareness of cognitive processes. Leitão et al. (2022) and Antonaci *et al.* (2019) showed that the integration of pedagogical content through the application of gamification principles, such as avatars, rewards, and immediate feedback, strengthens intrinsic motivation and fosters curiosity, which are the key aspects of meaningful and self-regulated learning. Critical perspectives on the use of gamification were also identified. As noted by Juan-Lázaro & Area-Moreira (2021) poorly structured gamified strategies, or those excessively focused on point accumulation, may promote a trial-and-error dynamic without deep reflection, thereby limiting the development of metacognitive processes by prioritizing immediate outcomes over conscious analysis and regulation of learning.

Likewise, the discussion is framed within explicit theoretical foundations. These findings confirm that gamification, when grounded in constructivist approaches, Self-Determination Theory, and Self-Regulated Learning Theory, facilitates the activation of metacognitive strategies by fostering autonomy, intrinsic motivation, and reflection ( Qiao et al., 2022). This integration demonstrates that psychological and pedagogical perspectives are essential for explaining why gamification strengthens planning, monitoring, and evaluation in the learning process.

However, studies indicate that instructional design processes find a suitable gamification scenario to evaluate the relationship between goal setting, the use of badges, scoreboards, missions, and cognitive challenges, and factors related to metacognition, such as awareness, self-assessment, goal setting, and decision-making, as scholars do not support the idea that gamification inherently generates metacognitive processes ( Juan-Lázaro & Area-Moreira, 2021; McGowan et al. 2023; Qiao et al. 2022).

In addition, the reviewed literature suggests that gamification fosters the development of higher-order thinking skills (HOTS) through progressive challenges, problem-solving dynamics, and immediate feedback, as students engage in processes of analysis, evaluation, and creation, which are characteristic of HOTS ( Qiao, 2024). A positive relationship between gamification and metacognition can be inferred if metacognitive awareness is related to the use of HOTS.

Gamification can thus become a key element in the development of pedagogical strategies with high potential for promoting metacognitive processes, which is why its implementation should be supported by explanatory and theoretical frameworks aligned with clear educational purposes. The pedagogical value of gamification lies in its ability to generate reflective learning experiences as students are required to consciously and autonomously engage in planning, monitoring, and evaluating their cognitive processes. Through the activation of self-regulation mechanisms, the stimulation of critical reflection, and the strengthening of higher-order thinking skills, students can incorporate dynamics of autonomous and meaningful learning ( Cáceres Reche et al., 2022; Hidayat et al., 2024; Zhao et al., 2024).

One limitation of this study was that the analysis was restricted to only two databases, which may have excluded relevant studies from other bibliographic sources. Furthermore, only open-access articles were reviewed, indicating a potential publication bias. Therefore, future systematic reviews are recommended to broaden the scope of the search by including other recognized databases as well as non-open-access articles.

Finally, the review shows that gamification should articulate three complementary axes: first, the learning methodologies that guide its implementation; second, the psychological foundations that explain the mechanisms of motivation, autonomy, and student engagement; and third, the metacognitive processes that directly support planning, monitoring, and evaluation of learning are related to these mechanisms. This connection between theory and practice reinforces the idea that gamification should be considered from a didactic perspective with strong potential to foster higher-order thinking skills (HOTS).

## 5. Conclusions and limitations

The findings of this study allow us to conclude that gamification, when implemented in a structured manner and supported by learning theories, constitutes a process that fosters the development of metacognitive skills ( García-López et al., 2023; Pirta-Dreimane et al., 2024). The reviewed studies showed that the incorporation of elements such as feedback, rewards, progressive challenges, and autonomy promotes skills such as planning, self-regulation, monitoring, and the evaluation of learning ( Edo Agustín, 2023). These skills are activated in gamified contexts, which in turn stimulates student engagement and conscious decision-making throughout the learning process.

This study also contributes to the educational field by synthesizing recent evidence and proposing an integrated perspective that links the analysis of the impact of gamification on the development of metacognitive processes by considering psychological learning theories, learning methodologies (especially GBL), and the development of metacognitive processes.

Similarly, this study highlights the need to understand its application in education from perspectives that integrate both the conceptual and procedural aspects. At the conceptual level, this study indicates that gamification should be understood based on a set of psychological theories that explain, on the one hand, what learning is and how it occurs, and, on the other, which cognitive processes are involved. At the procedural level, this study emphasizes the importance of approaching gamification from a didactic perspective by addressing questions related to the role of specific tools in teaching and learning content, skills, and competencies, as reflected in game-based learning (GBL). The results warned that the effectiveness of gamification depends on instructional design, as it is through design that principles of gamification can be effectively applied to foster the development of metacognitive skills.

Finally, this review reveals that gamification and metacognition have not yet been fully studied in educational contexts. It also underscores the need for a broader evaluation of the existing literature, given that the study was conducted solely with open-access articles from Scopus and Web of Science, which may have restricted the inclusion of other relevant studies.

## Ethics and consent

Ethical approval and consent were not required for this study, because it was a systematic review of previously published studies that did not involve human participants.

## Data Availability

All datasets supporting the findings of this systematic review are openly available in the Zenodo repository at **https://doi.org/10.5281/zenodo.17626131** ( Varela Quiroga & Buitrago Ropero, 2025). The repository contains the PRISMA 2020 Checklist and the PRISMA 2020 Flow Diagram. All materials are provided under a Creative Commons CC0 1.0 Universal license. These files ensure full reproducibility of the review methodology and comply with the F1000Research open data policy.
