# Supplementary material for: Gamification and the Development of Metacognitive Processes: A Systematic Literature Review
Source: F1000Res. 2026 Oct 13;14:1387. Originally published 2025 Dec 10. [Version 3] doi: 10.12688/f1000research.173134.3 (PMC13722855; doi:10.12688/f1000research.173134.3)
Supplement: Peer review history

# Reviewer report: Reviewer response for version 3

**DOI:** 10.5256/f1000research.211539.r529693

**Published:** 2026-10-30

**Authors:** Daniel Fernández Galeote

**Affiliations:** 1 Tampere University, Tampere, Finland

Thanks for your changes. The "EB" tab still has a date in Spanish and one of the other tabs even has the title in Spanish. If you can upload a new version to the repository, that would be great. However, this is only a minor point.

Are the rationale for, and objectives of, the Systematic Review clearly stated?

Partly

Is the statistical analysis and its interpretation appropriate?

Not applicable

If this is a Living Systematic Review, is the ‘living’ method appropriate and is the search schedule clearly defined and justified? (‘Living Systematic Review’ or a variation of this term should be included in the title.)

Not applicable

Are sufficient details of the methods and analysis provided to allow replication by others?

Partly

Are the conclusions drawn adequately supported by the results presented in the review?

Partly

Reviewer Expertise:

Games and gamification

I confirm that I have read this submission and believe that I have an appropriate level of expertise to confirm that it is of an acceptable scientific standard.

# Reviewer report: Reviewer response for version 2

**DOI:** 10.5256/f1000research.198005.r481373

**Published:** 2026-05-25

**Authors:** Daniel Fernández Galeote

**Affiliations:** 1 Tampere University, Tampere, Finland

Thank you for your improvements. Most of my questions have been answered, doubts have been cleared and certain limitations have been acknowledged.

 However, I still have issues understanding the supplementary file. There are column titles in Spanish, empty or inconsistent column contents, and a series of five research questions that, while useful, do not match the ones in the study itself. There is a richness of data there that is not in the study, and I wonder why; it almost looks like article and matrix follow two different logics altogether.

 In terms of content inconsistencies, I looked at a particular column that looked interesting to me (RQ2¿Cuáles son las herramientas de gamificación en línea que promueven el uso del desarrollo de procesos metacognitivos?) to find that sometimes discrete elements are listed, other times game titles are provided, and sometimes there are just entire sentences describing the games in an unsystematic way.

 For this matrix to be useful to readers, in my view it should be much more systematized and carefully revised. Given the high-level overview of the results in the article I treat this supplementary file as an important part of the article, not just a secondary add-on.

Are the rationale for, and objectives of, the Systematic Review clearly stated?

Partly

Is the statistical analysis and its interpretation appropriate?

Not applicable

If this is a Living Systematic Review, is the ‘living’ method appropriate and is the search schedule clearly defined and justified? (‘Living Systematic Review’ or a variation of this term should be included in the title.)

Not applicable

Are sufficient details of the methods and analysis provided to allow replication by others?

Partly

Are the conclusions drawn adequately supported by the results presented in the review?

Partly

Reviewer Expertise:

Games and gamification

I confirm that I have read this submission and believe that I have an appropriate level of expertise to confirm that it is of an acceptable scientific standard, however I have significant reservations, as outlined above.

# Response

**Published:** 2026-07-09

**Authors:** CLAUDIA VIVIANA VARELA QUIROGA

**Affiliations:** Cundinamarca, Universidad Antonio Narino, Bogotá, Bogota, Colombia

Thank you for your careful review and for emphasizing the importance of the supplementary material. We agree that the extraction matrix should be clear, systematic, and aligned with the manuscript. Following your suggestions, we thoroughly revised the supplementary file.

 Specifically:

1. Column titles in Spanish: All column headings have been translated and standardized in English.
2. Empty or inconsistent contents: We reviewed the  matrix, corrected formatting inconsistencies and typographical errors, standardized the terminology  and harmonized the entries across all records.
3. Five research questions versus three research questions: The previous version of the extraction matrix contained five preliminary research questions because it was originally developed as an internal data extraction instrument during the early stages of the review. As the systematic review progressed, the analytical framework was refined and consolidated into the three research questions presented in the final manuscript. The extraction matrix has now been revised to reflect the final research questions and analytical structure of the study.
4. Inconsistencies in the RQ2 column: We agree with your observation. This column was systematically revised to eliminate inconsistencies arising from mixing game titles, gamification elements, and descriptive statements. The information has now been organized using standardized and controlled categories consistent with the analytical framework adopted in the manuscript.
5. Alignment between the article and the supplementary file: We reviewed the extraction matrix to ensure that it follows the same analytical logic as the manuscript. In addition, we verified that the frequencies reported are supported by the updated extraction matrix.

The revised extraction matrix has been uploaded as a new version in Zenodo:

**https://zenodo.org/records/21270796**

 We sincerely appreciate your comments, which have substantially improved the clarity, consistency, and usefulness of the supplementary material

# Reviewer report: Reviewer response for version 2

**DOI:** 10.5256/f1000research.198005.r481374

**Published:** 2026-05-21

**Authors:** Erry Hidayanto

**Affiliations:** 1 Universitas Negeri Malang, Malang, Indonesia

I've reviewed the author's response and it's been revised. I agree with the corrections made.

Are the rationale for, and objectives of, the Systematic Review clearly stated?

Yes

Is the statistical analysis and its interpretation appropriate?

Yes

If this is a Living Systematic Review, is the ‘living’ method appropriate and is the search schedule clearly defined and justified? (‘Living Systematic Review’ or a variation of this term should be included in the title.)

No

Are sufficient details of the methods and analysis provided to allow replication by others?

Yes

Are the conclusions drawn adequately supported by the results presented in the review?

Yes

Reviewer Expertise:

NA

I confirm that I have read this submission and believe that I have an appropriate level of expertise to confirm that it is of an acceptable scientific standard.

# Reviewer report: Reviewer response for version 1

**DOI:** 10.5256/f1000research.190917.r443379

**Published:** 2026-01-21

**Authors:** Erry Hidayanto

**Affiliations:** 1 Universitas Negeri Malang, Malang, Indonesia

1. In the introduction, substantial gaps are not yet apparent.

 2. Regarding the methods, the number of reviews was unequal. Of the 253 reviewed, 181 were excluded, and 71 were analyzed until the end, resulting in a total of 252, not 253.

 3. The PRISMA model guidelines refer to Moher et al. (2009), while the latest standard is PRISMA 2020.

 4. The research results do not explicitly indicate that the findings address the RQ.

 5. The conclusion is still a theoretical summary, not a systematic synthesis.

 6. The conclusion does not reflect a pattern of evidence, but rather a causal relationship.

Are the rationale for, and objectives of, the Systematic Review clearly stated?

Yes

Is the statistical analysis and its interpretation appropriate?

Yes

If this is a Living Systematic Review, is the ‘living’ method appropriate and is the search schedule clearly defined and justified? (‘Living Systematic Review’ or a variation of this term should be included in the title.)

No

Are sufficient details of the methods and analysis provided to allow replication by others?

Yes

Are the conclusions drawn adequately supported by the results presented in the review?

Yes

Reviewer Expertise:

Notes can be seen in the script

I confirm that I have read this submission and believe that I have an appropriate level of expertise to confirm that it is of an acceptable scientific standard, however I have significant reservations, as outlined above.

# Response

**Published:** 2026-03-21

**Authors:** CLAUDIA VIVIANA VARELA QUIROGA

**Affiliations:** Cundinamarca, Universidad Antonio Narino, Bogotá, Bogota, Colombia

We sincerely thank Dr. Erry Hidayanto for the constructive and precise feedback. All comments have been carefully addressed, and the manuscript has been substantially revised to improve conceptual clarity, methodological accuracy, and analytical rigor. A detailed response is provided below.

 1. Introduction: research gap not clearly identified

 Comment: Substantial gaps are not yet apparent.

**Response:**

 The Introduction has been substantially revised to clarify the research gap. We now explicitly state that, although gamification has been widely studied in education, there is a lack of systematic understanding of how specific gamification elements are linked to metacognitive processes. Additionally, we highlight that many studies report positive outcomes without clearly specifying instructional mechanisms or metacognitive scaffolding, which justifies the need for this review.

 2. Numerical inconsistency in study selection

 Comment: The number of reviewed articles is inconsistent (253 – 181 = 72, not 71).

**Response:**

 We appreciate this observation. The inconsistency has been corrected. The manuscript now clearly reports that 72 full-text articles were assessed, one was excluded due to confirmed plagiarism, and 71 studies were included in the final analysis, ensuring full consistency across the PRISMA flow description.

 3. PRISMA version

 Comment: PRISMA 2009 was used instead of PRISMA 2020.

**Response:**

 This has been corrected. The manuscript now explicitly follows and references the PRISMA 2020 guidelines (Page et al., 2021) throughout the Methods section, including updated reporting of the selection process.

 4. Results do not explicitly answer research questions

 Comment: Findings do not clearly address the RQ.

**Response:**

 The Results section has been reorganized to explicitly align with each research question RQ1–RQ3. Each subsection now presents findings directly linked to the corresponding research question, improving coherence and analytical clarity.

 5. Conclusion is descriptive rather than synthetic

 Comment: The conclusion is still theoretical, not a systematic synthesis.

**Response:**

 The Conclusions section has been substantially revised to reflect a pattern-based synthesis of the evidence. Rather than summarizing concepts, we now identify recurring conditions under which metacognitive outcomes are reported, providing a more analytical and evidence-based synthesis.

 6. Causal interpretation of findings

 Comment: The conclusion reflects causal relationships rather than patterns of evidence.

**Response:**

 We have carefully revised the manuscript to avoid causal claims. The findings are now explicitly framed as associations conditioned by instructional design features, rather than direct causal effects. We emphasize that gamification does not inherently produce metacognitive development, but that outcomes depend on the intentional integration of reflective scaffolding and pedagogical alignment.

 We appreciate the reviewer’s valuable feedback, which has contributed to improving the conceptual clarity, methodological consistency, and analytical depth of the manuscript. We believe the revised version addresses all concerns and provides a more robust and transparent systematic review.

# Reviewer report: Reviewer response for version 1

**DOI:** 10.5256/f1000research.190917.r443386

**Published:** 2026-01-07

**Authors:** Daniel Fernández Galeote

**Affiliations:** 1 Tampere University, Tampere, Finland

- Article summary and overall opinion

 This study systematically reviews the relationship between gamification and metacognition, as well as wider higher-order thinking skills. The review is based on 71 articles from 2019-2024 found through Scopus and WoS. The findings indicate that gamification is associated with metacognition through a variety of elements and psychological processes, and its use to this end justified via a small number of theories and frameworks.

 I found the article easy to read and tackling an important topic. Because this journal focuses primarily on "the scientific validity of the article, rather than the novelty or interest levels", my observations below focus on framing and analytical limitations that I consider should be addressed to make this review as sound as it can be. My main concerns include a limited framing of concepts and research questions, unjustified choices, and limited systematicity in the reporting of findings, in addition to incomplete (as far as I saw, and if I am not mistaken) supplemental files. If these can be clarified and amended, the review can offer a valid contribution, but they should be seen as a request for rather significant revisions. Therefore, my decision assumes that the requests can be fulfilled by the authors.

 - General comments by section

 1. Introduction

 The section does a good job of summarizing the relationship between gamification and metacognition at a general level, even though this relationship is presented in an exclusively positive way: only the potential and evidence for the usefulness of gamification are discussed, but in my view there could be room for deeper questions at this point (e.g., where does gamification work and not work?) and possible limitations of this approach supporting the need for a critical systematic review. See, e.g., my and my colleagues' qualitative assessment of student experiences with gamified metacognitive prompts (https://doi.org/10.1145/3757980.3758006).

 One related comment is about this segment: "Despite this evidence, the implementation of gamification in educational institutions has hardly been explored, particularly regarding the analysis and study of its psychological and pedagogical frames, as well as its specific effect on the development of metacognition." I suggest rephrasing this sentence so that it is less ambiguous about what has been studied: education tends to be by far the most explored context of gamification (https://doi.org/10.1016/j.ijinfomgt.2018.10.013) and its psychological and pedagogical (theoretical) frames have been extensively studied (e.g., https://doi.org/10.1016/j.chb.2021.106963), as well as practical methods of application (too many design frameworks to even cite). If the issue here is that there is no systematic understanding of how gamification interacts with metacognition, I'd just go for that argument unambiguously.

 2. Materials and Methods

 RQ1 does not refer to the educational context only, but the search string limits the results to anything explicitly connected with education. Is this not a mismatch that renders RQ1 essentially inadequate for this review?

 The concept of HOTS (RQ3) was not clearly introduced and defined before. It was just said that metacognition is "one of them" but why is the study now focusing on these? If the review focuses on these, an expanded introduction explaining this concept, its significance, and why it can be included within the same review is needed.

 The time limit imposed (2019 on) is not explicitly justified.

 Why were "cognitive ability" and "awareness" chosen as the most relevant keywords to include besides metacognition? Is this based on trial searches? Existing metacognition frameworks such as https://doi.org/10.3991/ijet.v15i21.14907 and https://doi.org/10.3390/su15021668 would have probably suggested relevant key concepts to metacognition (e.g., self-regulation). Please justify the choices or acknowledge the limitations.

 I conducted a new search on Scopus for the period 2019-2024 using the same string. It yielded 337 documents versus the 165 resported, indicating that this area of research (at least in the way paraphrased in the search string used) is being constantly growing and updating, even within a restricted time frame. This may be worth mentioning in the limitations, since a reader cannot expect the results to be necessarily exhaustive of even the time frame chosen.

 Please consider rephrasing the following sentences, I do not understand the "corresponding database" part in the first sentence and the grammar in the second one: "During this phase, one article was excluded after verifying that it had been reported as plagiarized, because it was not part of the corresponding database. The exclusion criteria were ethical and scientific considerations were excluded from this study."

 "The following samples were extracted from each study: general bibliographic information, study-level metadata, methodological characteristics, gamification-related variables, metacognition-related variables, theoretical frameworks, higher-order cognitive skills, relevance to each research question, and main conclusions extracted from each study." Can you please provide more details on how analytical categories within each of these variables were formed and applied? Especially when talking about "methodological characteristics, gamification-related variables, metacognition-related variables, theoretical frameworks, higher-order cognitive skills", are these deductively created from pre-existing frameworks (which should be referenced) or inductively formed from the studies analyzed? If so, what was the process?

 "The risk of bias in the studies was evaluated using criteria adapted from qualitative evidence." I would need more detail about this to truly grasp what is meant. What qualitative evidence? In addition, this risk of bias seems to not appear again in the manuscript. How was it applied? For what?

 3. Results

 The answer to RQ1 is presented, as the introduction anticipated, in an exclusively positive way, but there are hints that gamification could not work as intended: for example, sources seem to talk about "the appropriate integration of game-based content" or "well-designed gamification". Therefore, it would be very interesting for the reader to know more about how "appropriate" and "well-designed" gamification looks. If these studies don't say, then that's a limitation worth discussing.

 "According to Leal Uhlig et al. (2023), technology plays a key role in GBL environments..." So did all the studies analyzed incorporate digital technology (I take "technology" to mean digital in this context)? This is somewhat surprising, since there are many GBL methods (board games, card games, role-playing games) that do not require digital technology at all.

 The fact that so many studies seemed not to specify their learning methodologies, learning theories or cognitive processes is surprising and should be discussed. Also, I think that self-determination theory should not be labelled as a "psychological theory of learning" since it is about human motivation. Consider changing the category's name.

 In general, the results are presented in a way that feels rather generic: examples of implementations are mentioned in an example-like, unsystematic way, and for the most part gamification is treated as a unitary concept. Yet, implementations vary very significantly and it is not clear to me how we can talk about gamification as a whole without doing a deeper analysis that attempts to systematically categorize different designs (e.g., based on game elements and affordances). One example of a sentence that hints at this problem is the following: "gamification, by integrating reflective strategies, such as self-ssessment journals and evaluation spaces, promotes awareness of one’s own learning processes". This really sounds like the methods that promote awareness are journals and evaluation spaces, which are not intrinsic to gamification nor do they require it. This is, at the very least, a limitation of this review. Alternatively, a systematic account of the mechanisms used in each study and a table presenting the relationship between methods, psychological processes and outcomes (sytematizing observations such as, e.g., "augmented reality shows that gamified activities involve exploration, analysis, and evaluation, which makes higher-order thinking skills (HOTS) more explicit in the learning process" or "solving challenges requires the application of thinking and logical skills (cognitive challenges), and often overcoming challenges that depend on solving previous situations, which implies a progression in the complexity of thinking") would greatly improve the contribution and the apparent soundness of the conclusions. The incompleteness of the supplemental files (see below) makes this crucial.

 A minor point: the figures have inconsistent fonts at times.

 4. Discussion

 "This systematic review explored the existing literature in relation to the influence of gamification on the development of metacognitive processes in educational settings" But what about HOTS?

 "Elements, such as feedback, progressive challenges, immediate rewards, and constant reflection stand out." Without a systematic presentation of elements, the reader cannot tell what "standing out" means. Is this a quantitative assessment?

 The discussion could be more critical in its appraisal of the subject; the closest is this sentence which resonates with some of my comments above: "scholars do not support the idea that gamification inherently generates metacognitive processes (Juan-Lázaro & Area-Moreira, 2021; McGowan et al. 2023; Qiao et al. 2022)". My requests should help provide a more structured answer to the question of how gamification can generate metacognitive processes and what pitfalls may exist.

 The discussion includes a couple of limitations but section 5 is the one labelled "limitations". Please also consider multiple additional limitations mentioned throughout my peer review.

 "Finally, the review shows that gamification should articulate three complementary axes: first, the learning methodologies that guide its implementation; second, the psychological foundations that explain the mechanisms of motivation, autonomy, and student engagement; and third, the metacognitive processes that directly support planning, monitoring, and evaluation of learning are related to these mechanisms. This connection between theory and practice reinforces the idea that gamification should be considered from a didactic perspective with strong potential to foster higher-order thinking skills (HOTS)." If you mean here that articles should clarify these more explicitly, as I too mentioned above, please say so. Aside from this, the paragraph refers to a "connection between theory and practice" but I don't understand what it means. The authors chose these three axes themselves, so they established the connection: if you mean that the findings of the review reinforce the existence of this connection, please be more clear about it.

 5. Conclusion and Limitations

 "this study highlights the need to understand its application in education from perspectives that integrate both the conceptual and procedural aspects. At the conceptual level, this study indicates that gamification should be understood based on a set of psychological theories that explain, on the one hand, what learning is and how it occurs, and, on the other, which cognitive processes are involved. At the procedural level, this study emphasizes the importance of approaching gamification from a didactic perspective by addressing questions related to the role of specific tools in teaching and learning content, skills, and competencies, as reflected in game-based learning (GBL)." I agree, and as I said above, it would be excellent if this review itself could be more systematic about the way in which it presents these conceptual and procedural aspects of gamification, rather than mostly narrative.

 "this review reveals that gamification and metacognition have not yet been fully studied in educational contexts" How so? What part of the findings revealed this?

 Supplemental files

 The data extraction matrix linked in Zenodo appears to me severely incomplete: most of the analytical categories are missing from it and it still includes the "plagiarized" article. Please provide a full matrix.

Are the rationale for, and objectives of, the Systematic Review clearly stated?

Partly

Is the statistical analysis and its interpretation appropriate?

Not applicable

If this is a Living Systematic Review, is the ‘living’ method appropriate and is the search schedule clearly defined and justified? (‘Living Systematic Review’ or a variation of this term should be included in the title.)

Not applicable

Are sufficient details of the methods and analysis provided to allow replication by others?

Partly

Are the conclusions drawn adequately supported by the results presented in the review?

Partly

Reviewer Expertise:

Games and gamification

I confirm that I have read this submission and believe that I have an appropriate level of expertise to confirm that it is of an acceptable scientific standard, however I have significant reservations, as outlined above.

## References

[1] : The rise of motivational information systems: A review of gamification research. *International Journal of Information Management* .2019;45: 10.1016/j.ijinfomgt.2018.10.013191-210 10.1016/j.ijinfomgt.2018.10.013

[2] : Revealing the theoretical basis of gamification: A systematic review and analysis of theory in research on gamification, serious games and game-based learning. *Computers in Human Behavior* .2021;125: 10.1016/j.chb.2021.106963 10.1016/j.chb.2021.106963

[3] : The 8 Pillars of Metacognition. *International Journal of Emerging Technologies in Learning (iJET)* .2020;15(21) : 10.3991/ijet.v15i21.14907 10.3991/ijet.v15i21.14907

[4] : Meta-Learning: A Nine-Layer Model Based on Metacognition and Smart Technologies. *Sustainability* .2023;15(2) : 10.3390/su15021668 10.3390/su15021668

# Response

**Published:** 2026-03-21

**Authors:** CLAUDIA VIVIANA VARELA QUIROGA

**Affiliations:** Cundinamarca, Universidad Antonio Narino, Bogotá, Bogota, Colombia

We sincerely thank Dr. Daniel Fernández Galeote for the thoughtful and constructive feedback, which has significantly contributed to improving the clarity, rigor, and analytical depth of the manuscript. All comments have been carefully addressed in the revised version. Below, we provide a point-by-point response.

 1.  Introduction: conceptual framing and critical perspective

 Comment: The introduction presents gamification in an exclusively positive way and lacks a critical perspective.

**Response:**

 We have revised the Introduction to incorporate a more balanced and critical perspective. Specifically, we now acknowledge that the effects of gamification are not uniform and depend on instructional design quality, contextual alignment, and the explicit inclusion of metacognitive scaffolding. We also clarified that the gap addressed by this review is not the absence of research on gamification in education, but the lack of a systematic understanding of how gamification interacts with metacognitive processes.

 2.  Research questions and conceptual clarity (HOTS)

 Comment: HOTS was not clearly introduced or justified.

**Response:**

 We expanded the theoretical framing of Higher-Order Thinking Skills (HOTS) in the Methods section, explicitly defining the construct and justifying its inclusion. We clarified that HOTS were included because several studies examined metacognition alongside broader cognitive outcomes, and that metacognitive regulation is frequently conceptualized as a mechanism underlying these skills.

 3.  Methodological transparency

 Comment: Lack of justification for keywords, time frame, and search decisions.

**Response:**

 We improved methodological transparency by:

 •   Justifying the time range (2019–2024) as reflecting recent developments in gamified educational practices and digital integration.

 •   Explaining the inclusion of keywords such as “cognitive ability” and “awareness” as overlapping constructs used to capture metacognitive processes.

 •   Explicitly acknowledging the limitations of the search strategy, including potential omissions of relevant literature.

 4.  Data extraction and analytical process

 Comment: Lack of clarity on how analytical categories were formed.

**Response:**

 We clarified that the analytical categories were primarily developed through an inductive thematic synthesis, based on recurring patterns identified in the included studies. We also described how the coding process was iteratively refined and validated through reviewer consensus.

 5.  Risk of bias and quality appraisal

 Comment: Insufficient explanation and unclear use.

**Response:**

 We clarified that, due to methodological heterogeneity, a formal risk-of-bias tool was not applied. Instead, we conducted a qualitative appraisal of reporting transparency, theoretical grounding, and alignment between design and outcomes. This limitation is now explicitly acknowledged in the manuscript.

 6.  Results: lack of systematicity and overgeneralization

 Comment: Results are presented in a generic way.

**Response:**

 We substantially revised the Results section to:

 •   Emphasize the heterogeneity of gamification designs, avoiding treating gamification as a single unified construct.

 •   Clarify that reported outcomes depend on specific design features.

 7.  Mechanisms and instructional design

 Comment: Lack of clarity on what “well-designed” gamification means.

**Response:**

 We addressed this by identifying recurring instructional features associated with metacognitive outcomes, including:

 •   Structured feedback

 •   Goal-setting tasks

 •   Opportunities for reflection and self-assessment

 We also explicitly stated that many studies do not sufficiently operationalize these elements, which is now discussed as a key limitation.

 8.  Discussion: need for critical analysis

 Comment: Discussion lacks critical depth.

**Response:**

 The Discussion has been strengthened to:

 •   Avoid causal claims and instead frame findings as conditional associations

 •   Highlight limitations in the evidence base

 •   Emphasize that gamification does not inherently produce metacognition, but depends on intentional instructional design

 9.  Conclusions: need for systematic synthesis

 Comment: Conclusions were too general and not evidence-based.

**Response:**

 We revised the Conclusions to:

 •   Reflect patterns of evidence across studies

 •   Avoid overgeneralization

 •   Emphasize conditions under which metacognitive outcomes are more likely to occur

 10. Supplemental files

 Comment: The matrix was incomplete.

**Response:**

 We have revised and updated the data extraction matrix to ensure completeness and alignment with the analytical categories described in the manuscript. The updated dataset is now available in the repository.

 We appreciate the reviewer’s thoughtful and constructive comments, which have contributed to improving the conceptual clarity, methodological transparency, and analytical rigor of the manuscript. We believe the revised manuscript addresses the concerns raised and provides a more robust and systematic contribution to the field.
